# Assessment of Temporal Somatosensory Discrimination in Females with Fibromyalgia: Reliability and Discriminative Ability of a New Assessment Tool

**DOI:** 10.3390/s24113300

**Published:** 2024-05-22

**Authors:** Christophe Demoulin, Léonore Jodogne, Charline David, Jean-François Kaux, Marc Vanderthommen

**Affiliations:** 1 Department of Physical Activity and Rehabilitation Sciences, University of Liege, 4000 Liege, Belgium; 2 Department of Physical Medicine and Rehabilitation, University Hospital Centre, 4000 Liege, Belgium; 3 Faculty of Motor Sciences, Université Catholique de Louvain-La-Neuve, 1348 Louvain, Belgium

**Keywords:** somatosensory discrimination, fibromyalgia syndrome, chronic pain, tactile acuity, reliability, discriminative ability

## Abstract

We assessed the test–retest reliability and discriminative ability of a somatosensory temporal discrimination (SSTD) assessment tool for fibromyalgia syndrome (FMS) and determined if pain-related variables were associated with SSTD performance. Twenty-five women with FMS and twenty-five asymptomatic women were assessed during two sessions 7 to 10 days apart. The proportion of correct responses (range 0–100) was calculated. Sociodemographic information was collected for both groups. The participants with FMS also completed the widespread pain index and the Brief Pain Inventory. Test–retest reliability was verified by calculating intraclass correlation coefficients. Discriminative ability was verified by a between-group comparison of scores using a *t*-test. Associations between SSTD score and pain variables were tested using Pearson or Spearman correlation coefficients. The test–retest reliability of the SSTD score was excellent (ICC > 0.9, CI: 0.79–0.96) for the asymptomatic group and good for the FMS group (ICC: 0.81, 95% CI: 0.62–0.91). The median (Q1–Q3) test session SSTD score differed significantly between the FMS 84.1 (71–88) and the asymptomatic 91.6 (83.4–96.1) groups (*p* < 0.001). Only pain duration was associated with the SSTD score. In conclusion, the new SSTD test seems reliable for people with FMS and is discriminative. Further studies should examine its sensitivity to change and correlations with other SSTD tests.

## 1. Introduction

Fibromyalgia syndrome (FMS) is characterised by diffuse, chronic, widespread pain and symptoms such as fatigue, sleep disorders, cognitive dysfunctions, and mood disturbances [1]. FMS is believed to be related to changes in the central nervous system and immunological activity [2,3] caused by an increase in central sensitisation and an impairment in pain processing [4,5]. Cortical reorganisation associated with chronic pain can lead to changes in the person’s image of their own body and a loss of somatosensory discrimination (SSD) [6,7,8]. SSD, or tactile acuity, is the ability to distinguish between two tactile stimuli. It is generally assessed using the two-point discrimination test [9,10], which focuses on spatial discrimination. Somatosensory temporal discrimination (SSTD) is the perception of two distinct stimuli applied separately at short intervals [11]. SSTD involves the activation of the somatosensory cortex and striatal neurons [12] and is thought to reflect the reorganisation of the somatosensory cortex more strongly than spatial SSD [13]. Both types of discrimination involve central and peripheral neuronal mechanisms, but central phenomena are thought to predominate in temporal SSD [14]. Furthermore, SSTD seems to depend on inhibitory mechanisms within the primary somatosensory area (S1) [14], which may be impaired in chronic pain [15].

Several studies have evaluated SSTD [11,13,16,17,18], but only one focused on people with FMS [11]. It suggested that SSTD capacity is correlated with pain and disability scores [11]. The authors suggested these findings could be related to a cortical reorganisation of the somatosensory cortex, disturbance of self-awareness, or cognitive disturbances with impaired attention–focusing. Some evidence suggests that training tactile acuity in people with chronic pain could reduce pain [7] and increase function [19]. Furthermore, divided attention tasks directed at two distinct areas of the body [20] and localised body-focused attention [21,22] appear to reduce pain; therefore, including attention flexibility tasks in the test appears relevant. Based on these findings, a novel gamified technology for remotely delivered somatosensory training in people with chronic pain was developed. The system trains pain modulation via an attentional focus on non-painful stimuli. It uses vibrotactile pods to deliver vibrational stimuli, rather than the more commonly used electrical stimuli, which are not always comfortable for individuals with pain syndromes. In a pilot study, we found that this system was feasible for use in people with FMS and resulted in high engagement, satisfaction, and adherence [23].

The primary aims of this study were to examine the test–retest reliability and ability to discriminate symptomatic individuals with a diagnosis of FMS in asymptomatic individuals through a new SSTD test. The secondary aim was to determine associations between SSTD scores and pain variables in people with FMS.

## 2. Materials and Methods

### 2.1. Design

We used a repeated measures design to determine the test–retest reliability of the SSTD test. We included a group of people with FMS and a group of asymptomatic people without FMS to determine the discriminative ability of the SSTD test. 

All the participants were informed of the objective of the project and consented to their participation. The study was approved by the Liege University Hospital Human Ethics Committee (29 March 2021: B7072021000013).

The guidelines for reporting reliability and agreement studies (GRRAS) were followed [24].

### 2.2. Participants

Participants were recruited through advertisements placed in hospitals, healthcare clinics, and the social media accounts of some FMS associations. Inclusion criteria for the symptomatic group were women aged 18–70 years with a diagnosis of FMS made by a specialist physician using the 2016 criteria [25]. We excluded people who self-rated their average pain intensity over the previous week as <3/10 on a numerical rating scale.

Inclusion criteria for the asymptomatic group were females aged 18–70 years with no diagnosis/history of FMS and no history of chronic pain. 

Exclusion criteria for both groups were difficulty understanding instructions (or French) or an intellectual deficit, pregnancy or in the post-partum period, diagnosis of a neurological disease (stroke, epilepsy, or peripheral neuropathy) that may affect the somatosensory system, and having participated in the somatosensory rehabilitation programme we previously proposed using this technology, or participation in the testing conducted during the development of the somatosensory temporal discrimination assessment application in the past year.

Asymptomatic participants were included after the inclusion of the participants with FMS in order to match them (i.e., position the vibrotactile pods in similar zones in both groups, see below).

### 2.3. Evaluation Sessions

All the participants underwent a test session and a retest session 7 to 10 days later, conducted by one of two final-year physiotherapy students who received specific training for this purpose. The same person supervised the test and retest sessions for a given participant.

The test session included a general questionnaire, clinical questionnaires, a pain intensity evaluation, an explanation of the SSTD test, a familiarisation phase, and the SSTD test. The retest session was identical except that the familiarisation phase was omitted, and the general questionnaire was replaced by a question about whether a particular event had occurred since the last session that might have altered the person’s somatosensory discrimination capacity and/or pain status (e.g., change in medication).

### 2.4. Assessments

Participants could either complete the questionnaires online just prior to attending the session or at the beginning of the session.

The general information questionnaire asked about demographic information and professional situations. The FMS participants were asked to mark their area of greatest pain among prespecified areas on a body chart (posterior shoulders/trapezius, anterior shoulders, thoracic spine/scapula/ interscapular area, lumber area, hips, or knees). This information was used to determine the position of the vibrotactile pods for the SSTD test.

Pain and its characteristics were evaluated using the widespread pain index (WPI) (FMS participants only) and the Brief Pain Inventory (BPI) (all participants). 

The WPI assesses the degree of pain in 19 body locations (e.g., shoulder girdle left, shoulder girdle right, chest, neck, lower back, etc.) over the past week. We used the French version [26]. One point is attributed to each area marked; the points are summed to yield a total score ranging from 0 to 19, with higher scores indicating more widespread pain. This tool is also used to diagnose FMS; we did not use the section relating to diagnosis since all FMS participants already had a medical diagnosis of the condition [27]. 

The BPI assesses pain intensity (severity) and the impact of pain on functioning (interference) [28,29]. We used the French version [30]. It assesses pain intensity using 4 numerical pain scales ranging from 0 (no pain) to 10 (maximum pain), including the highest and lowest pain intensities during the previous week, average pain levels, and pain at the time of the assessment. The BPI also contains 7 items exploring the impact of the person’s pain on their daily life (i.e., general activity, walking, work, mood, enjoyment of life, relations with others, and sleep). To score the BPI, the assessor calculates the average of the four severity items (pain severity subscale) and the average of the seven interference items (pain interference subscale). 

The pain intensity in the area designated as the area of greatest pain was evaluated using a numerical rating scale (0–10 points) (FMS participants only).

### 2.5. Temporal Somatosensory Discrimination (SSTD) Test 

**Figure 1:**
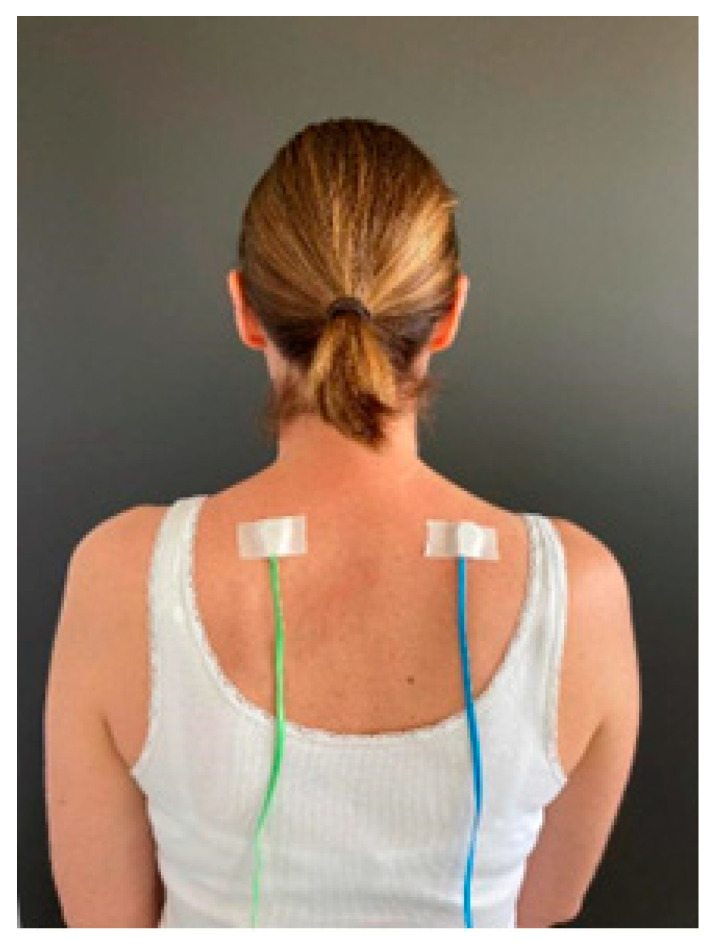
Example of vibrotactile pod placement on both sides of the body.

**Figure 2:**
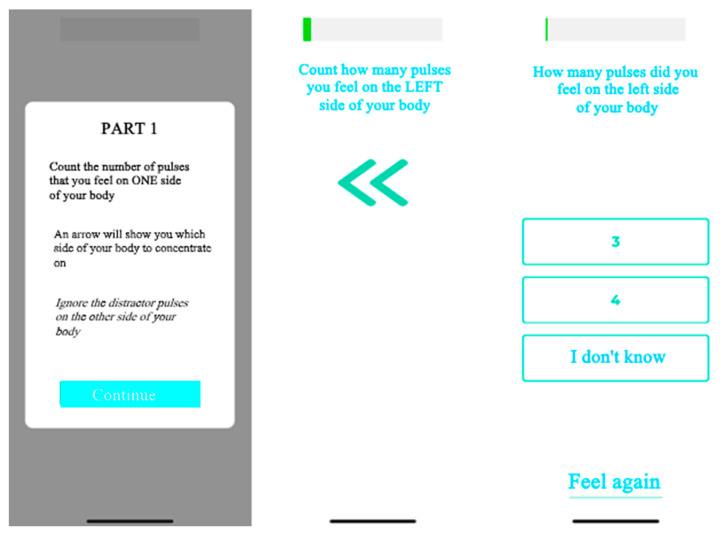
Example of the application used for the SSTD test.

The SSTD device (TrainPain Inc., Wilmington, DE, USA) consists of a box linked by wires to 2 vibrotactile pods that are operated via Bluetooth by a smartphone application. The same device can be used to train and assess SSTD. One vibrotactile pod was positioned on the most painful area indicated by the participant and the other on the same area on the contralateral side of the body (Figure 1). Measurements were taken with reference to anatomical landmarks to ensure that the vibrotactile pods were repositioned in the same places at the retest session. Asymptomatic participants were matched to FMS participants, and the vibrotactile pods were positioned in the same places. The participants sat in a chair with care taken to ensure the vibrotactile pods were not in contact with the chair. They wore earplugs to ensure they did not hear the vibrations. The vibrotactile pods on the tested side of the body provided three or four consecutive vibration trails, while the contralateral vibrotactile pod provided distractor vibrations. The test was divided into four different phases, with a 3-min rest in between, and included 112 trials (28 for each phase). These four phases differed regarding the stimulation modalities, i.e., the time between pulses of the vibrotactile pods on the tested side and the strength/intensity of the pulses of the distraction pods. All the instructions were provided by the application on the phone screen (e.g., “count how many vibrations you feel on the RIGHT (or LEFT) side of your body” accompanied by an arrow indicating the side concerned). Once the vibrations had been emitted, the screen changed and offered several response options: the participant had to indicate the number of vibrations they felt on the side indicated, and they could also ask to ‘feel again’ (maximum twice per phase) or select ‘I don’t know’ (Figure 2). 

The outcome analysed was the SSTD score, which corresponded to the success rate on the SSTD test, calculated as the proportion of correct responses out of the total number of responses. Therefore, the lowest possible score was 0 and the highest was 100.

A familiarisation phase was performed prior to the test. It consisted of 10 trials similar to those at the end of phase 1 (the easiest trials, i.e., with long intervals between pulses and the lowest distractor intensity). The participants were informed that the test would also include some trials with slightly different characteristics.

### 2.6. Statistical Analysis

Regarding the sample size, our target was to include 25 patients with FMS and 25 matched asymptomatic participants. This pragmatic sample size was based on recruitment feasibility and staff capacity (considering this was a non-funded study).

Statistical analyses were carried out using Excel and R commander version 4.2.2 statistical software. 

The data distribution was verified using the Shapiro–Wilk test for each variable. 

The participant demographic characteristics and SSTD scores were compared between groups using the parametric Student’s *t*-test or the Mann–Whitney U test, as appropriate. The chi-square test was used to compare laterality and professional situations between groups.

Test and retest SSTD scores were compared using a paired Student *t*-test. The reliability of the SSTD score for each group and the total sample was assessed using the intra-class correlation coefficient, ICC 3.1, and calculation of the standard error of measurement (SEM): SD × √ (1−ICC).

In the FMS group, correlation analyses were performed by calculating Pearson or Spearman correlation coefficients (depending on the normality of the distributions). The variables included were duration of pain, pain intensity in the area of greatest pain, and BPI and WPI scores.

A *p*-value < 0.05 was considered significant for all the analyses.

## 3. Results

### 3.1. Participants

Twenty-five participants with FMS and twenty-five asymptomatic participants were included. All the participants performed both sessions. Their characteristics are shown in Table 1. The groups did not differ in terms of age or laterality, however, they differed significantly in terms of professional situation; significantly fewer people in the FMS than the asymptomatic group were currently working.

### 3.2. Test–Retest Reliability (Table 2)

The test–retest reliability was excellent (ICC > 0.9) for the asymptomatic group and good for the FMS group (0.75 < ICC < 0.9). The SEM was slightly higher in the FMS than in the asymptomatic group. The SSTD performance did not differ between the sessions for either group. 

No participants had a score of 0; therefore, there was no floor effect. There was also no ceiling effect, as the overall score of 100% was only achieved by two participants, both in the FMS group.

**Table 2:** Test–retest reliability.

	Test	Retest	*p*-Value	ICC	95% CI	SEM
FMS (N = 25)	84.1 (71–88) [61.4–100]	80.6 (72.7–92.6) [67.5–100]	0.67	0.81	0.63–0.92	5.4
Asymptomatic (N = 25)	91.6 (83.4–96.1) [48.8–99]	92.5 (86.2–95.1) [47.8–99]	0.85	0.90	0.80–0.96	3.6
Total (n = 50)	NA	NA	0.21	0.86	0.77–0.92	4.6

Data are the median (Q1–Q3) [min–max]. SEM: standard error of measurement, FMS: fibromyalgia syndrome, NA: not applicable.

### 3.3. Discriminative Ability

The median (Q1–Q3) test session scores of the FMS 84.1 (71–88) and the asymptomatic 91.6 (83.4–96.1) groups differed significantly (*p* < 0.001).

### 3.4. Associations between Discrimination Performance and Pain Variables in the FMS Group (Table 3)

Only the duration of pain in years was significantly correlated with the SSTD score. Pain intensity, BPI scores, and WPI were not. 

**Table 3:** Correlations between SSTD performance and pain variables.

	r	*p*-Value
Duration of pain (years)	−0.41	<0.001
Pain intensity in the area of vibrotactile pod placement	0.12	0.22
BPI (Severity)	−0.09	0.39
BPI (Interference)	−0.08	0.4
WPI	−0.02	0.8

## 4. Discussion

The present study is one of the first to determine the test–retest reliability of an SSTD test and the first to investigate the reliability of such a test conducted on the most painful body parts in people with FMS. To our knowledge, this is the only test that does not involve electrical stimuli. The test is original in its use of a distractor stimulus that tests inhibition capacity. The results showed good test–retest reliability of the SSTD score, particularly in the asymptomatic group. The results of the asymptomatic and SFM groups differed significantly, indicating the test’s discriminative ability. The only variable associated with SSTD performance in the FMS group was pain duration. 

In a preliminary study by our group (unpublished data), we observed a learning effect of the test. Therefore, we included a familiarisation phase, which appeared to eliminate the learning effect since no significant differences were found between the test and the retest sessions. Furthermore, test–retest reliability was excellent for the asymptomatic group and good for the FMS group (0.75 < ICC < 0.9). This is higher than the test–retest reliability found over three test sessions in a group of healthy adults using electrical stimulation, in which the ICC values showed moderate to good reliability [31]. The SEM of 3.6 for the asymptomatic group and 5.4 for the FMS group in our study suggest that the minimal detectable [32] change is 10 and 15 in these groups, respectively. 

The SSTD score of the participants with FMS was significantly lower than that of the asymptomatic participants, demonstrating the discriminative ability of the SSTD test. One participant in the asymptomatic group had a particularly low score (test = 48.8, retest = 47.8) for unknown reasons. Without this participant, the between-group difference would have been even larger. These results are in line with the current literature, confirming the impairment of SSD in people with chronic pain [5,6,11,17,19]. The only other study that compared the SSTD threshold between people with FMS and healthy individuals also found a difference between people with and without FMS; however, they only tested the hand and used an electrical stimulus [11]. The alteration in SSTD ability in people with FMS might result from changes in the primary somatosensory cortex and a reduction in inhibitory processes [14]. Nevertheless, five of the twenty-one participants with FMS had mean scores that were above the median score of the asymptomatic participants, indicating that not all individuals with FMS have impaired SSTD. Furthermore, two participants (both professionally active) with FMS achieved 100% scores. These findings suggest that SSTD capacity should be assessed prior to proposing a re-training programme to ensure that such a program is relevant for the individual. It might also be relevant to increase the difficulty of the test to enable changes to be evaluated in people with good initial scores if such training is relevant for them. 

A significant negative correlation was found between the duration of pain symptoms and the SSTD score. The influence of pain duration on cortical representations is well known. Grey matter in the areas of the cortex involved in central sensitisation reduces in the case of chronic pain, and the magnitude of the reduction is related to pain duration and intensity [33]. Therefore, SSTD may be an indicator of cortical reorganisation over time, although this needs to be confirmed. However, in contrast with a study that included only 15 individuals with FMS [11], we found no correlations between performance on the SSTD test and pain and disability scores. The different SSTD assessment method and pain/disability outcomes used in that study, and the younger age (mean 33 years) and shorter duration of symptoms (6.8 years) of participants, limit comparison. Further studies should be conducted to investigate such links.

The plasticity that underlies pain-related changes in the brain suggests that these changes may be sensitive to targeted treatments [5]. SSD training induces cortical reorganisation, improved tactile acuity, and reduced pain in people with chronic pain or phantom pain [8,19,34,35,36]. Attentional modulation, which involves the descending and ascending pain pathways, is thought to be altered in people with chronic pain [3,37]. Therefore, various therapeutic techniques use attentional focus to reduce pain, such as cognitive-behavioural therapy, yoga, meditation, hypnosis, and relaxation [37]. Besides the assessment mode, the tool investigated here provides a new means of pain modulation through attentional processes by focusing on non-painful stimuli during SSTD training [23].

Assessing SSD in people with chronic pain with this new, original tool that enables remote assessment, administered independently by the individual, is therefore relevant and useful from both clinical and research points of view. Although the spatial form of SSD is most often assessed using the two-point discrimination test [10], this new approach may be more representative of central neurological phenomena [13,16] and thereby more relevant for the assessment of people with central sensitisation. Another special feature of this new SSTD testing system is the inclusion of a distractor stimulus that must be ignored. This forces the person to shift their attention in space (from one side of the body to the other), thus enabling exploration of attentional processes. These processes, also trained during mindfulness meditation [21], might be beneficial for people with chronic pain. Furthermore, this new system can be used by the individuals themselves, without a healthcare professional, thus the test can be performed by telehealth. 

Although the above elements highlight the relevance of the present study, it has several limitations. As only female participants were included, our results cannot be generalized to males. Some demographic characteristics (e.g., marital status and BMI) were not recorded, preventing us from fully verifying the similarities of the groups. 

Further studies should now investigate if SSTD training with this new system improves SSTD capacity and if these improvements are related to improvements in pain, disability, and/or quality of life of people with FMS, particularly those with low SSTD capacity. It would also be relevant to test the validity of this new system by investigating if SSTD performance correlates with other SDT tests. 

## 5. Conclusions

This study found good to excellent test–retest reliability of the SSTD test in both people with FMS and asymptomatic participants and confirmed its ability to discriminate between these two groups. Only the duration of pain correlated with SSTD performance. SSTD performance was heterogeneous across participants; therefore, this test might be used to identify individuals with reduced SSTD who may benefit from an intervention. The next step is to determine the sensitivity to change in the SSTD test to ensure it can be used to evaluate the effectiveness of training programs.

## Figures and Tables

**Table 1:** Demographic characteristics of all participants and pain-related scores for the FMS group.

	FMS (N = 25)	Asymptomatic (N = 25)	*p*-Value
Age (years), mean (SD) [min–max]	51.4 (9.5) [24–65]	49.1 (6.2) [37–60]	0.27
Laterality			
Right-handed	24 (96%)	32 (92%)	0.55
Left-handed	1 (4%)	2 (8%)	
Professional situation			0.0001
Retired	24% (n = 6)	0% (n = 0)	
Unemployed	4% (n = 1)	0% (n = 0)	
Sick leave	40% (n = 10)	0% (n = 0)	
Working	32% (n = 8)	100% (n = 25)	
Duration of pain (years)	14.9 (9.56) [1–38]	-	
BPI, mean (SD) [min–max]			
Pain severity score (0–10) Pain interference score (0–10)	5.84 (1.77) [2.25–9.5] 5.47 (1.89) [2.14–9.7]	-	
WPI (0–19)	13.1 (4.16) [5–19]	-	
Most painful area and location of the vibrotactile pods			
Shoulders (posterior/trapezius)	44%	-	
Lumbar	20%	-	
Hips	20%	-	
Knees	8%	-	
Thoracic area	8%	-	
Shoulders (anterior)	0%	-	

FMS: fibromyalgia syndrome; BPI: Brief Pain Inventory; WPI: widespread pain index.

## Data Availability

The dataset is available on request from the authors.
